# Clinical Note on Preparing Solution of Lacto-Somatose

**Published:** 1899-08

**Authors:** 


### CLINICAL NOTE ON PREPARING SOLUTION OF LACTO-SOMATOSE.

Lacto-somatose is now generally considered as a prominent food and restorative in the treatment of gastro-intestinal diseases attended with marked exhaustion, due to the drain of fluids from the system and the marked impairment of the digestive functions. Hence, a few remarks as to the manner of conveniently preparing solutions might not be out of place. The simplest way of dissolving the preparation is to place the desired dose upon the surface of about one-quarter glassful of hot water and allow it to dissolve without stirring. This will require about ten to fifteen minutes, when a clear solution of agreeable taste and appearance is obtained, which may be drank in its own form or mixed with ordinary fluids, such as milk, soups, gruels, etc. Another simple means of dissolving lacto-somatose is to sprinkle it upon hot milk or broths with a dry wire sieve. A still other method is to stir it into a smooth paste with a little water before adding the remainder of the solvent, but this may be conveniently replaced by the above methods. The chief points to which attention has been particularly directed with reference to the therapeutics of lacto-somatose comprise its large percentage of assimilable albumins, rendering it a prompt and efficient tonic and food; its beneficial action upon the digestive tract, stimulating the appetite and relieving vomiting, and its freedom from taste and odor enabling it to be added to a variety of beverages, so that it never excites repugnance, even when administered for long periods.—*Clinical Notes*.

